# Supplementary figures and images for: The Mbam drainage system and onchocerciasis transmission post ivermectin mass drug administration (MDA) campaign, Cameroon
Source: PLoS Negl Trop Dis. 2021 Jan 19;15(1):e0008926. doi: 10.1371/journal.pntd.0008926 (PMC7815102; doi:10.1371/journal.pntd.0008926)

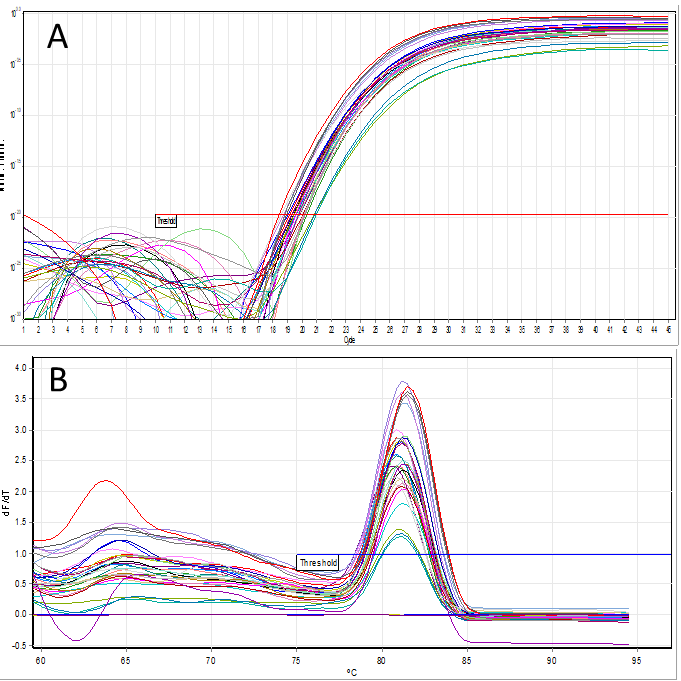

Supplement: S1 Fig — (TIF) [file pntd.0008926.s003.tif]

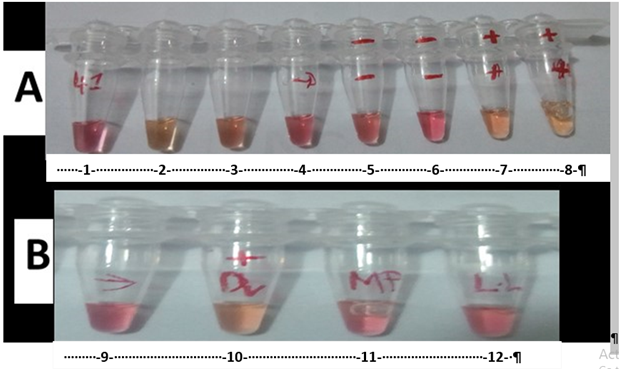

Supplement: S2 Fig — A.). Samples containing O. volvulus larval stage(s) turned yellow and were scored positive (i.e. tubes -2-, -3-, -7- and -8-). Negative pools remained pink (i.e. tubes -1-, -4-, -5- and -6-). B.). Specificity of colorimetric LAMP assay confirmed. Reaction contained no template DNA (> or tube -9-) or DNA from Onchocerca volvulus (OV or tube -10-), or Mansonella perstans (MP or tube -11-) or Loa loa (LL or tube -12-). (TIF) [file pntd.0008926.s004.tif]
