# Supplementary material for: The Mbam drainage system and onchocerciasis transmission post ivermectin mass drug administration (MDA) campaign, Cameroon
Source: PLoS Negl Trop Dis. 2021 Jan 19;15(1):e0008926. doi: 10.1371/journal.pntd.0008926 (PMC7815102; doi:10.1371/journal.pntd.0008926)
Supplement: Attachment — Submitted filename: PNTD-D-20-01257 - Mbam River Drainage review.docx [file pntd.0008926.s006.docx]

PNTD-D-20-01257:Observations on the hydrologic network and dynamics of onchocerciasis transmission within nine months following a mass drug administration campaign with ivermectin in the Mbam river drainage, a site with persistent transmission in Cameroon

This is an important paper that should be published. It shows the importance of moving from control to elimination of onchocerciasis. This area has been under treatment for more than 20 years, and yet the impact of an annual dose of ivermectin has not interrupted transmission. The only point I would like to raise for the authors to consider is that Mbam transmission zone is not limited to Centre Region of Cameroon. It seems to extend into other areas including Massangam Health District of West Region around R. Nja, a tributary of R.Noun, R.Kichi, a tributary of R. Mbam (Katabarwa, Eyamba, Nwane et al, 2013). Therefore, success can only be attained if the limits of the entire transmission zone are properly delineated and interventions such as ground larviciding and community directed doxycycline are applied.
