# Supplementary material for: The Mbam drainage system and onchocerciasis transmission post ivermectin mass drug administration (MDA) campaign, Cameroon
Source: PLoS Negl Trop Dis. 2021 Jan 19;15(1):e0008926. doi: 10.1371/journal.pntd.0008926 (PMC7815102; doi:10.1371/journal.pntd.0008926)
Supplement: Attachment — Submitted filename: MANUSCRIPT REVIEW FOR PLOS NEGLECTED TROPICAL DISEASES.doc [file pntd.0008926.s007.doc]

**MANUSCRIPT REVIEW FOR PLOS NEGLECTED TROPICAL DISEASES**

**NAME AND ADDRESS OF REVIEWER:**

**Prof. B. E. B. Nwoke, FAS**

**Professor of Public Health Entomology & Parasitology**

Member, Nigerian Steering Committee Neglected Tropical Diseases (NTDs)

Chairperson, Nigerian Onchocerciasis Elimination Committee

Member, Mectizan Expert Committee

Department of Animal & Environmental Biology

**IMO STATE UNIVERSITY, OWERRI**

**NIGERIA.**

Telephone: (+234) 80 33 27 39 15

**E-mail:** [**bebndie@yahoo.com**](mailto:bebndie@yahoo.com)

**TITLE:** Observations on the hydrologic network and dynamics of onchocerciasis transmission within nine months following a mass drug administration campaign with ivermectin in the Mbam river drainage, a site with persistent transmission in Cameroon

**MANUSCRIPT NUMBER**: PNTD-D-20-01257

**REVIEWER’S COMMENTS**

**1. Title:** The title is good, relevant and operational but I suggest that it can be made smarter by reframing as **“Hydrologic network and onchocerciasis transmission post ivermectin MDA campaign in the Mbam river drainage, Cameroon”**

**2. Abstract;** Consistent with expected standard but the following corrections on **RED** can be effected to add advantage to it:

**Introduction:** The impact of large scale Mass Drug Adminstration (MDA) of ivermectin on *Simulium damnosum*, which transmits the parasite *Onchocerca volvulus* is of great importance for onchocerciasis control programmes. We investigated the impact of MDA of ivermectin on entomological indices and also verify if there are hydrologic network factors that could have favoured the transmission of onchocerciasis in this area and contribute to the persistence of disease. We compared three independent techniques to detect Onchocerca larvae in blackflies and also analyzed the hydrologic network within 9 months post**-**MDA of ivermectin.

**Method:** Methodology is consistent with expected standard and the experimental design is satisfactory.

**Results: Satisfactory but the underlisted correction should be effected:**

- Line 49: We used human landing collection method (previously called human bait) to collect 22,274 adult female *Simulium* flies from Mba River System. Of this number, 9,134 were dissected while 129 pools constituted for

**Conclusion:** **Satisfactory but the under listed corrections should be effected:**

- Line 56: Results from fly dissection (Microscopy), real-time PCR and LAMP revealed the same trends
- **Key words:** Mbam river drainage, MDA Ivermectin Simulium, biting rate, infection rate

**2. Introduction: The under listed corrections should be effected:**

- **Line 82-83:** Onchocerciasis is a parasitic disease caused by *Onchocerca volvulus,* transmitted by the bite of an infective female
- **Line 92:** The control/elimination of onchocerciasis today relies on ivermectin (Mectizan**^®^**) mass drug administration
- **Line 95:** programmes. Mectizan® has been donated free of charge by Merck and Co.
- Line 96/97: Whitehouse Station, NJ since 1987. Ivermectin must be repeatedly given once a year for more than 15 years (with at least 60% geographical and chemotherapeutic coverage of MDA) to successfully achieve the elimination of onchocerciasis by the year 2030
- **Line 128/129:** Following the adoption of IVM-MDA as a control/eliminaltion strategy in Cameroon, it has been demonstrated that the Community Directed Treatment with Ivermectin (CDTI) project of Central Region at the level of Bafia had limited impact on
- **Line 134:** Delete (or Mf are not being cleared in the treated individuals)
- **Line 137:** vector capacity and efficiency of *Simulium damnusum* for the transmission of onchocerciasis in this area and
- **Lines 139-145:** Should be reframed to reflect the objectives of the study not the procedure of study

**3. STUDY AREA: Effect the following correction:**

- **Lines 170-177:** The geographical and chemotherapeutic coverage of the MDA in the past 15 years of the transmission zone should be included
- **Line 181:** Collection of *Simulium* flies by human landing method
- **Lines 207 – 272** covering the following should be reframed and summarized.

(i) Genomic DNA extraction from pools of *Simulium* flies,
(ii) (ii) Detection of *O. volvulus* in pool-DNA of blackfly using colorimetric O-150 LAMP and
(iii) Detection of *O. volvulus / O. ochengi* in pool-DNA of blackfly using real-time PCR

**4. RESULT:** Presentation of the result is satisfactory and consistent with expected standard. However, the results of the revised manuscript should factor in the geographical and chemotherapeutic coverage of the MDA in the past 15 years of the transmission zone

**5. DISCUSSION:** The result of this study is well discussed but the absence of the data on the impact of geographical and chemotherapeutic coverage of the MDA in the past 15 years on the transmission zone should be taken into consideration.

6. CONCLUSION: There is need for the introduction of the geographical and chemotherapeutic coverage of the MDA in the past 15 years of the transmission zone and its impact on the dynamics of *Onchocerca volvulus* infection in the black flies in the conclusion.

**7. REFERENCES:** The authors demonstrated an in-depth knowledge of the field of study and also made due acknowledgement of existing information. However, there are minor collections that should be effected:

(a) The biological names in reference numbers 20, 25, 30, 35, 36 and 38 should be in Italics
(b) Reference 32 should re-written to be consistent with the expected format.

**8. OBSERVATIONS**

(a) This study has made original contributions to scientific knowledge by bringing to focus the impact of hydrologic network/characteristics of transmission zone and other factors on onchocerciasis transmission post-mass MDA of ivermectin
(b) The introduction of the geographical and chemotherapeutic coverage of the MDA in the past 15 years of the transmission zone and its impact on the dynamics of *Onchocerca volvulus* infection in the black flies is going to add value to the work.

**9. RECOMMENDATIONS**

(a) The manuscript should **be accepted** for publication **but NOT in its present form**
(b) The corrections pointed out should be effected before publications, especially the **introduction of the treatment coverage (both geographical and chemotherapeutic coverage) of the MDA** in the past 15 years of the transmission zone and its impact on the dynamics of *Onchocerca volvulus* infection

Thanks for inviting me to serve and contribute.

Please accept the continued assurances of my highest esteem and regards.

**Prof. B. E. B. Nwoke**
