# Supplementary material for: The Mbam drainage system and onchocerciasis transmission post ivermectin mass drug administration (MDA) campaign, Cameroon
Source: PLoS Negl Trop Dis. 2021 Jan 19;15(1):e0008926. doi: 10.1371/journal.pntd.0008926 (PMC7815102; doi:10.1371/journal.pntd.0008926)
Supplement: Attachment — Submitted filename: Abong et al. Responses to Reviewers.doc [file pntd.0008926.s008.doc]

**A letter containing a point-by-point response to the reviewer comments and a description of the changes made in the revised version of manuscript**

Date: September 22, 2020

**The Editors-in-Chief and the Editorial Board of,**

**PLOS Neglected Tropical Diseases**

Dear Dr. Stolk WA, Dr. Hellemond JV and the Editorial Board,

We are very grateful for the thorough review done on our manuscript (PNTD-D-20-01257). We have addressed all the concerns of the reviewers. We have also indicated all changes made on the revised manuscript with track changes.

Yours sincerely.

**Samuel Wanji, PhD**

Corresponding author.

**Reviewer reports and Responses to reviewers’ comments**

We are very grateful to the reviewer comments. The responses to their comments and observations are in blue colour in this document and changes made in the manuscript are highlighted in the revised version with track changes.

**Reviewer #1**

We did not see any specific comments/suggestions/observations to be addressed from this reviewer.

**Reviewer #2:** Prof. B. E. B. Nwoke

| **Comment/Observation/Suggestion** | **Response** |
| --- | --- |
| The results of the revised manuscript should factor in the geographical and chemotherapeutic coverage of the MDA in the past 15 years of the transmission zone | The geographical and therapeutic coverages of this area have been reported in other studies. The coverages have been included in the study area section (Lines 167-173) as follow;  In this transmission zone, MDA coverage from inception of CDTI in the year 2000 up to the time of this study have improved steadily over the years as reported earlier . As of the year 2007, WHO reported a 99.5% geographical coverage of all 15 CDTI projects in Cameroon including the Centre 1 CDTI project where the Mbam drainage basin is situated. The therapeutic coverage in the Centre 1 CDTI project increased from as low as 42% in 2000 to above 75% in 2009, ranged between 75-80% from 2010 to 2014 and remained above 80% during the 2015, 2016 and 2017 treatment periods . |
| There is need for the introduction of the geographical and chemotherapeutic coverage of the MDA in the past 15 years of the transmission zone and its impact on the dynamics of *Onchocerca volvulus* infection in the black flies in the conclusion. | This has been included in the conclusion (lines 557-563) as follows;  Despite the reported high geographical (99.5%) and improved therapeutic (from 42% to over 80%) coverages of MDA in this area over the years, the high vector abundance (enhanced by the river drainage system) with high vector capacity and efficiency have been able to continuously transmit the parasite from the few infected individuals among those who do not comply to treatment (or the few individuals carrying parasite strains that respond sub-optimally to ivermectin) to non-infected individuals, to maintain the observed uninterrupted transmission profile before and after MDA campaign. |
| Title: The title is good, relevant and operational but I suggest that it can be made smarter by reframing as “Hydrologic network and onchocerciasis transmission post ivermectin MDA campaign in the Mbam river drainage, Cameroon” | Thank the reviewer for his suggestion on the title. We agree with him that the suggested title is more concise and smarter. We have effected the change but replaced “Hydrologic network” with “Mbam drainage system” as pointed out by reviewer #3. (lines 2 and 3).  The new title reads “**The Mbam drainage system and onchocerciasis transmission post ivermectin Mass Drug Administration (MDA) campaign, Cameroon”** |
| Abstract; Consistent with expected standard but the following corrections on RED can be effected to add advantage to it:  **Introduction:** The impact of large scale Mass Drug Adminstration (MDA) of ivermectin on *Simulium damnosum*, which transmits the parasite *Onchocerca volvulus* is of great importance for onchocerciasis control programmes. We investigated the impact of MDA of ivermectin on entomological indices and also verify if there are hydrologic network factors that could have favoured the transmission of onchocerciasis in this area and contribute to the persistence of disease. We compared three independent techniques to detect *Onchocerca* larvae in blackflies and also analyzed the hydrologic network within 9 months post-MDA of ivermectin. | We have effected the corrections in RED following the suggestion of the reviewer (lines 38-46) |
| **Results:** Satisfactory but the underlisted correction should be effected:  ♣    Line 49: We used human landing collection method (previously called human bait) to collect 22,274 adult female *Simulium* flies from Mbam River System. Of this number, 9,134 were dissected while 129 pools constituted for | The corrections have been effected (lines 53-55). |
| **Conclusion:** Satisfactory but the under listed corrections should be effected:  ♣    Line 56: Results from fly dissection (Microscopy), real-time PCR and LAMP revealed the same trends | The correction has been effected (line 62) |
| **♣    Key words:** Mbam river drainage, MDA Ivermectin *Simulium*, biting rate, infection rate | The key words have been reduced to “Mbam river drainage, MDA Ivermectin, *Simulium*, biting rate, infection rate” as suggested. (lines 66-67) |
| **Introduction:** The under listed corrections should be effected:  ♣    Line 82-83: Onchocerciasis is a parasitic disease caused by *Onchocerca volvulus*, transmitted by the bite of an infective female | The correction has been effected (line 89) |
| ♣    Line 92: The control/elimination of onchocerciasis today relies on ivermectin (Mectizan®) mass drug administration | The correction has been effected (line 98) |
| ♣    Line 95: programmes. Mectizan® has been donated free of charge by Merck and Co. | The correction has been effected (line 101) |
| ♣    Line 96/97: Whitehouse Station, NJ since 1987. Ivermectin must be repeatedly given once a year for more than 15 years (with at least 60% geographical and chemotherapeutic coverage of MDA) to successfully achieve the elimination of onchocerciasis by the year 2030 | The correction have been effected (lines 102-104) |
| ♣    Line 128/129: Following the adoption of IVM-MDA as a control/eliminaltion strategy in Cameroon, it has been demonstrated that the Community Directed Treatment with Ivermectin (CDTI) project of Central Region at the level of Bafia had limited impact on | We have effected the corrections (lines 135-137) |
| ♣    Line 134:  Delete (or Mf are not being cleared in the treated individuals) | It has been deleted (lines 141-142) |
| ♣    Line 137: vector capacity and efficiency of *Simulium damnusum* for the transmission of onchocerciasis in this area and | The correction has been effected (line 145) |
| ♣    Lines 139-145: Should be reframed to reflect the objectives of the study not the procedure of study | The section has been reframed to reflect the study objectives (lines 148-154) as follows;  In order to know the best diagnostic tool for monitoring infection in vectors, we compared 3 independent diagnostic techniques namely: microscopy, real-time polymerase chain reaction (PCR) and loop-mediated isothermal amplification (LAMP) for the detection of *Onchocerca* larvae in blackflies within the nine months post IVM-MDA. We also wanted to understand the Mbam River drainage system and report on the dynamics of water flow at different seasons of the year. |
| **STUDY AREA:** Effect the following correction:  ♣    Lines 170-177: The geographical and chemotherapeutic coverage of the MDA in the past 15 years of the transmission zone should be included | The geographic and chemotherapeutic coverage of the MDA in the past years have been included (lines 166-171) as follows;  In this transmission zone, MDA coverage from inception of CDTI in the year 2000 up to the time of this study have improved steadily over the years as reported earlier . As of the year 2007, WHO reported a 99.5% geographical coverage of all 15 CDTI projects in Cameroon including the Centre 1 CDTI project where the Mbam drainage basin is found. The therapeutic coverage in the Centre 1 CDTI project increased from as low as 42% in 2000 to above 75% in 2009, ranged between 75-80% from 2010 to 2014 and remained above 80% during the 2015, 2016 and 2017 treatment periods . |
| ♣    Line 181: Collection of *Simulium* flies by human landing method | This correction has been effected (line 197) |
| ♣    Lines 207 – 272 covering the following should be reframed and summarized.  (i)    Genomic DNA extraction from pools of *Simulium* flies,  (ii)    (ii) Detection of *O. volvulus* in pool-DNA of blackfly using colorimetric O-150 LAMP and  (iii)    Detection of *O. volvulus* / *O. ochengi* in pool-DNA of blackfly using real-time PCR | We have reframed and summarised as suggested (lines 223-289). |
| **RESULT:** The results of the revised manuscript should factor in the geographical and chemotherapeutic coverage of the MDA in the past 15 years of the transmission zone | The geographical and therapeutic coverages of the MDA in the past 15 years of this transmission zone have been reported in other studies and also included in the study area section of this manuscript (line 167-173). Information on the impact of the coverages on transmission have also been discussed. |
| DISCUSSION: The result of this study is well discussed but the absence of the data on the impact of geographical and chemotherapeutic coverage of the MDA in the past 15 years on the transmission zone should be taken into consideration. | We have included information on the impact of geographical and chemotherapeutic coverage of the MDA in the discussion (lines 492-493, 502-505 and 515-520). |
| **CONCLUSION:** There is need for the introduction of the geographical and chemotherapeutic coverage of the MDA in the past 15 years of the transmission zone and its impact on the dynamics of *Onchocerca volvulus* infection in the black flies in the conclusion. | The geographical and therapeutic coverage of the MDA in the past years and its impact on the dynamics *O. volvulus* transmission have been introduced in the conclusion (lines 562-570) as follows;  “Despite the reported high geographical (99.5%) and improved therapeutic (from 42% to over 80%) coverages of MDA in this area over the years, the high vector abundance (enhanced by the river drainage system) with high vector capacity and efficiency have been able to continuously transmit the parasite from the few infected individuals among those who do not comply to treatment (or the few individuals carrying parasite strains that respond sub-optimally to ivermectin) to non-infected individuals, to maintain the observed uninterrupted transmission profile before and after MDA campaign.” |
| **REFERENCES:** The authors demonstrated an in-depth knowledge of the field of study and also made due acknowledgement of existing information. However, there are minor corrections that should be effected:  (a)    The biological names in reference numbers 20, 25, 30, 35, 36 and 38 should be in Italics  (b)    Reference 32 should re-written to be consistent with the expected format. | We have effected the corrections in the biological names (*italicised* them) in the reference list  Reference number 32 of Nzene *et al*. (2005) , now reference number 34 has been re-written properly. |
|  |  |

**Reviewer #3:**

| **Comment/Observation/Suggestion** | **Response** |
| --- | --- |
| Line 36;..Impact of large scale ivermectin MDA on *S. damnosum*……. This is as though Ivermectin had direct impact on *S. damnosum*…In that sense, there would be need to have entomological proof. Instead, it would be….Impact of large scale ivermectin MDA on active onchocerciasis transmission. Your investigations were based on the presence of *O. volvulus* larvae in the fly, and rather not the physical health of the fly. | The sentence has been corrected (lines 38-39) to read;  The impact of large scale Mass Drug Adminstration (MDA) of ivermectin on active onchocerciasis transmission by *Simulium damnosum,…* |
| Line 65;.. The presence of parasite strains that respond sub-optimally to an approved drug….With regular ivermectin MDA, its known that host parasite load is significantly reduced and consistent ivermectin MDA can cut oncho transmission even amidst abundance of *S. damnosum* biting….So,. ivermectin is not sub-optimally effective against microfilaria……..To dispute ivermectin, investigate and show proof of community compliance to ivermectin uptake. | We agree with the reviewer that regular ivermectin MDA significantly reduce host parasite load and consistent ivermectin MDA can cut onchocerciasis transmission even where there is abundance of vector. However, different studies have reported sub-optimal response to ivermectin in some foci either due to genetic drift as published by Doyle *et al*. in their study of genome-wide analyses which revealed genetic variation that significantly differentiated good responder (GR) and sub-optimal responder (SOR) *O. volvulus* parasites in samples from Cameroon and Ghana or due to long term MDA campaigns (multiple ivermectin treatments) as faster than expected skin repopulation rate with microfilaria or earlier than expected productivity by adult female *O. volvulus* worms after standard dose treatment have been reported. |
| Line 69;... The hydrologic network factors that could have favored abundance vector breeding and contributed to the persistence of disease transmission were also examined within the study period….I think what you exactly did here was a study of the mbam river drainage system. | Yes, we actually studied the Mbam drainage system (or Mbam river system) but thought the terms could be used interchangeably with hydrologic network. We have corrected it by replacing hydrologic network with the river system (or the Mbam drainage system) throughout the manuscript. We have also included Figure 7, which shows the view of the Mbam River at Bafia (Biatsotsa) during the dry season with an important water discharge (S3_video) as well as the presence of larval stages of vector on vegetation in the river (lines 454-456 and 461-465). |
| Line 92; The control of onchocerciasis today relies on ivermectin mass drug administration (IVM-MDA) to at risk populations in endemic countries….If this is true then ivermectin is not sub-optimally effective against micro filarial | There are many studies which show that IVM is effective but because of drug pressure, suboptimal responses to the drug by microfilaria have been reported . |
|  |  |

**References**
