# Supplementary material for: The Mbam drainage system and onchocerciasis transmission post ivermectin mass drug administration (MDA) campaign, Cameroon
Source: PLoS Negl Trop Dis. 2021 Jan 19;15(1):e0008926. doi: 10.1371/journal.pntd.0008926 (PMC7815102; doi:10.1371/journal.pntd.0008926)
Supplement: Attachment — Submitted filename: A letter containing a detailed list of responses_Oct._2020.doc [file pntd.0008926.s009.doc]

**A letter containing a detailed list of responses to the editorial recommendations, and a description of the changes made in the manuscript (PNTD-D-20-01257R1).**

Date: October 22, 2020

Dear Dr. Wilma A. Stolk (Associate Editor),

Dr. Jaap van Hellemond (Deputy Editor), and

The Editorial Board, PLOS Neglected Tropical Diseases

We are very grateful for the thorough review done on our manuscript (PNTD-D-20-01257R1). We have done the modifications according to the editorial recommendations. We have also indicated all changes made on the second revised manuscript with track changes.

Thank you for giving us the opportunity to resubmit a second revised version of this manuscript.

Yours sincerely.

**Samuel Wanji, PhD**

Corresponding author.

**Editorial recommendations and Responses to them**

We thank the editorial board for the recommendations. The responses to the recommendations are in blue colour in this document and changes made in the manuscript are highlighted in the second revised manuscript with tracked changes highlighted.

| **Editorial recommendations** | **Responses** |
| --- | --- |
| 1). References 3 and 5 are no longer available online. Please replace by more up to date references. | Thanks to the Editorial board for all the observations.  Both references (3 and 5) have been replaced with a more up to date one and the sentence ending with reference 5 modified to read as “The Global Burden of Disease Study estimated in 2017 that, there were 20.9 million prevalent *O. volvulus* infections worldwide with 14.6 million of them suffering from skin disease and 1.15 million had vision loss ”. **Lines 85-88.** |
| 2) Abstract, line 37: make clear that this work is done in the Mbam river system area | We have improved on the sentence to make clear that the work is done in the Mbam rivers system area.  The sentence now reads “We investigated in the Mbam river system area, the impact of ... (**Lines 36-37**). |
| 3) Methods, study area, lines 164-166: clarify how coverage was calculated in this case. If I am correct, you are referring to reported coverage levels, expressed as number treated out of the total population. Ref 24 also highlights that survey coverage estimates are considerably lower than the reported ones. This should be acknowledged. Check whether similar changes are required in the concluding paragraph of the paper. | We have clarified how the coverage was calculated in lines **163-164** …“(expressed as number treated out of the total population) …”  A statement has also been included to acknowledge that surveyed therapeutic coverage estimate in 2014 was considerably lower than that reported, as follows; “However, Kamga and colleagues observed a lower therapeutic coverage than that reported during the 2014 MDA campaign in the study site ”. **Lines 165-167.** |

**References**
